# Prognostic implications of type and density of tumour-infiltrating lymphocytes in gastric cancer

**DOI:** 10.1038/sj.bjc.6604738

**Published:** 2008-10-21

**Authors:** H E Lee, S W Chae, Y J Lee, M A Kim, H S Lee, B L Lee, W H Kim

**Affiliations:** 1 Department of Pathology, Seoul National University Bundang Hospital, Seongnam, Korea; 2 Department of Pathology, Kangbuk Samsung Hospital, Sungkyunkwan University School of Medicine, Seoul, Korea; 3 Department of Pathology, Seoul National University College of Medicine, Seoul, Korea; 4 Cancer Research Institute, Seoul National University College of Medicine, Seoul, Korea

**Keywords:** stomach neoplasms, tumour-infiltrating lymphocyte, immunohistochemistry, survival analysis, tissue array analysis

## Abstract

The study aims to determine whether type and density of tumour-infiltrating lymphocytes (TILs) can predict the clinical course in gastric cancer. Gastric carcinomas (*n*=220) were immunostained for CD3, CD8, CD20, and CD45RO and evaluated for clinicopathologic characteristics. Number of TILs that immunostained positively for each marker were counted using NIH ImageJ software. Tumours were grouped into low- and high-density groups for each marker (CD3, CD8, CD45RO). The densities of CD3^+^, CD8^+^, and CD45RO^+^ TILs were found to be independent predictors of lymph node metastasis by multivariate analysis with odds ratios (95% CI) of 0.425 (0.204–0.885), 0.325 (0.150–0.707), and 0.402 (0.190–0.850), respectively. Kaplan–Meier survival analysis revealed that patients in the high-density groups for CD3, CD8, and C45RO had a significantly longer survival time than the patients in the corresponding low-density groups, respectively. In multivariate survival analysis, the densities of CD3^+^, CD8^+^, and CD45RO^+^ TILs remained independent prognostic factors with hazard ratios (95% CI) of 0.549 (0.317–0.951), 0.574 (0.347–0.949), and 0.507 (0.298–0.862), respectively. In conclusion, density of TILs was found to be independently predictive of regional lymph node metastasis and patient survival in gastric cancer.

Despite a decreasing trend in its incidence, gastric cancer remains one of the most frequent causes of cancer-related death worldwide; more than 930 000 new cases are diagnosed and 700 000 deaths occur each year (Parkin *et al*, 2005). At present, surgical resection is considered the most reasonable procedure (Engstrom *et al*, 1985; Coombes *et al*, 1990), and tumour-node-metastasis (TNM) stage (by UICC/AJCC) assessed after resection is viewed as the prognostic factor that is most strongly associated with patient survival. However, it is not rare that gastric cancer patients with the same TNM stage pursue different clinical courses. Histopathologic classifications including WHO classification (Fenoglio-Preiser *et al*, 2000), Lauren's classification (Lauren, 1965), Ming's classification (Ming, 1977), and Goseki classification (Goseki *et al*, 1992) and molecular classifications (Hippo *et al*, 2002; Chen *et al*, 2005) have also been applied for the prediction of patient survival, but their prognostic accuracies are controversial (Lewin and Appleman, 1996; Fenoglio-Preiser *et al*, 2000). In addition, many attempts have been made to link molecular events in cancer cells with patient outcome, but none of these have been accepted to be clinically meaningful. In consequence, new prognostic determinants in conjunction with the TNM stage are required to predict patients' clinical course more reliably and precisely.

As the cancer immunosurveillance hypothesis was first proposed, the concept that the immune system can recognise and eliminate tumour cells has been energetically debated. Many experimental rodent studies on transgenic and knockout mice, and on monoclonal antibodies specific for distinct immunologic components have provided substantial evidence for the existence of cancer immunosurveillance, and in particular, have shown that the immune system indeed functions to protect murine hosts against development of both chemically induced and spontaneous tumours (Dunn *et al*, 2004). Moreover, in humans, epidemiologic data indicate that immunocompromised patients have a higher probability of developing cancers of both viral and non-viral origin, which supports the cancer immunosurveillance concept (Dunn *et al*, 2004). In addition, an accumulating evidence shows a positive correlation between the presence of lymphocytes in tumour tissue and increased patient survival.

Recent studies have shown that several types of tumour-infiltrating lymphocytes (TIL) are associated with better disease outcome for various human cancers, including melanoma (Clemente *et al*, 1996; Taylor *et al*, 2007) and colorectal (Pages *et al*, 2005; Galon *et al*, 2006), ovarian (Zhang *et al*, 2003), cervical (Piersma *et al*, 2007), hepatocellular (Gao *et al*, 2007), and urothelial (Sharma *et al*, 2007) carcinoma. These reports demonstrate that high numbers of CD3^+^, CD8^+^, or CD45RO^+^ T cells in tumour tissue are significantly correlated with lower frequencies of lymph node metastasis or disease recurrence, or longer patient survival. Moreover, Galon *et al* (2006) advocated that the type, density, and location of immune cells in colorectal cancer have prognostic values that are superior to and independent of those of the TNM classification . However, the effect of TILs on the clinical course of gastric cancer patients is largely unknown.

The aim of this study was to assess the prognostic role of TILs in gastric cancer. Here, we determined whether type and density of TILs can predict the regional lymph node metastasis and patient survival in gastric cancer.

## Materials and methods

### Patients and specimens

The files of 274 surgically resected consecutive gastric carcinoma cases examined at the Department of Pathology, Seoul National University Hospital between January and June 1995 were obtained to quantify TILs by immunohistochemistry using a tissue array method. Cases with cancer confined to mucosa were excluded because they have an excellent prognosis regardless of TIL status. Finally 220 tumour tissue samples were included in the analysis. Age, sex, histologic type (according to Lauren and WHO classifications), lymphatic invasion, and pTNM (pathologic TNM) stage were evaluated by reviewing medical records or the glass slides. Patient clinical outcomes were followed from the date of surgery until either the date of death or 31 December 2003, which resulted in a follow-up period that ranged from 1 to 108 months (mean, 64.4 months). The data of patients lost to follow-up and of those who died from a cause other than gastric cancer were regarded as censored during the survival analysis. No patient had received preoperative chemotherapy, and patients with stage II, III, and IV had received postoperative chemotherapy using fluorouracil (5-FU) -based regimen (5-FU alone, 5-FU plus mitomycin C, or 5-FU plus cisplatin). No patient had received pre or postoperative radiotherapy. This study was approved by the Institutional Review Board for Human Subject Research at Seoul National University Hospital.

### Tissue array method

Core tissue biopsies (2 mm in diameter) were taken from individual paraffin-embedded gastric carcinomas (donor blocks) and re-arranged in a new recipient paraffin block (tissue array block) using a trephine apparatus (Superbiochips Laboratories, Seoul, Korea). Six array blocks containing a total of 274 cases were prepared. As it has been shown that excellent staining result agreements are obtained for different intratumoral areas of gastric carcinomas (Lee *et al*, 2001), a core was sampled from each of the cases. Each paraffin block contained internal controls consisting of non-neoplastic gastric mucosa from the body, antrum, and intestinal metaplasia. Sections (4 *μ*m) were taken from each tissue array block, deparaffinised, and dehydrated.

### Immunohistochemistry

Formalin-fixed and paraffin-embedded sections were dewaxed in xylene, rehydrated through graded alcohol, and placed in an endogenous peroxide block for 15 min. Antigen retrieval was done using a microwave in 10 mM citrate buffer or using an autoclave in Borg solution (Biocare Medical, Walnut Creek, CA, USA). Non-specific staining was blocked by treating sections with 1% horse serum in Tris-buffered saline (pH 6.0) for 3 min. Anti-CD3 (total T-cell marker; microwave; 1 : 100; rabbit polyclonal; DAKO, Glostrup, Denmark), -CD8 (cytotoxic T-cell marker; autoclave in Borg solution; 1 : 100; Rabbit monoclonal, SP16; Neomarkers, Fremont, CA, USA), -CD20 (total B cell marker; microwave; 1 : 300; Mouse monoclonal, L26; DAKO), and -CD45RO (memory T-cell marker; microwave; 1 : 100; Mouse monoclonal, OPD4; Neomarkers) antibodies were then applied, and antibody binding was detected using an avidin–biotin–peroxidase complex (Universal Elite ABC Kit; Vectastain, Burlingame, CA, USA) for 10 min. Diaminobenzidine tetrahydrochloride solution (Kit HK153-5K; Biogenex, San Ramon, CA, USA) was then used as a chromogen.

### Quantitative analysis of TILs

Immunostained slides were scanned with a digital virtual microscope (dotSlide, Olympus, Hamburg, Germany), and a portion (area of 0.44 mm^2^ of tumour centre) of the virtual images was captured using an image viewer programme (OlyVIA, downloaded from http://www.olysia.co.kr/olyvia2992.zip) per case. The captured images were 1263 × 932 pixel in size and had a resolution of 1.633 pixel *μ*m^−1^ at a magnification of × 100. Numbers of positively immunostained cells per in the 0.44 mm^2^ target areas were counted using NIH imageJ software with a Java-based colour deconvolution plugin (http://rsb.info.nih.gov/ij).

### Statistical analysis

Comparisons between groups were performed using the Pearson's *χ*^2^ test or Fisher's exact (2-sided) test. Multivariate logistic regression was used to identify clinical and pathologic features predictive of regional lymph node metastases. Survival curves were estimated using the Kaplan–Meier product-limit method, and the significances of differences between survival curves were determined using the log-rank test. Multivariate comparisons of survival distributions were made using Cox proportional hazards models. All statistical analyses were conducted using SPSS 15.0 (SPSS, Chicago, IL, USA), and *P-*values of ⩽0.05 were considered statistically significant.

### Two-fold cross-validation approach

To dichotomise prognostic variables, CD3, CD8, and CD45RO, a two-fold cross-validation methodology and a minimum *P-*value approach (Mazumdar *et al*, 2003) were used. The dataset was randomly divided into two subsets. The inner 70% of the distribution from a marker was chosen as a selection interval of an optimal cutoff value. Stratified univariate and multivariate tests (logistic regression and Cox proportional hazards model) were performed for significance of prognostic variables.

## Results

### Density of TILs

**Figure 1:**
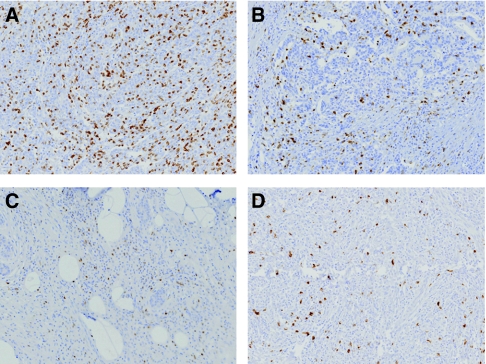
Types of tumour-infiltrating lymphocytes. CD3 (**A**), CD8 (**B**), CD45RO (**C**), and CD20 (**D**) expressing cells were detected immunohistochemically. They are tumour-infiltrating total T cells, cytotoxic T cells, memory T cells, and B cells, respectively (original magnification, × 100).

Tumour-infiltrating lymphocytes were detected within cancer epithelium or in stroma except for CD20^+^ TILs, which were mostly present in stroma (Figure 1). Mean numbers of CD3^+^, CD8^+^, CD45RO^+^, and CD20^+^ TILs per area of 0.44 mm^2^ were 353.87±381.55, 191.72±265.69, 76.13±138.30, and 89.42±152.45, respectively. Numbers of CD3^+^, CD8^+^, and CD45RO^+^ TILs were correlated with each other (range of correlation coefficients, 0.739–0.868; *P*<0.001), and numbers of CD20^+^ TIL were positively correlated with those of other TIL subtypes, but correlation coefficients were relatively low (range, 0.527–0.641; *P*<0.001). First, using mean number, all cases were classified into low- and high-density groups for each marker, that is, CD3_L_, CD8_L_, and CD45RO_L_ (low-density groups) and CD3_H_, CD8_H_, and CD45RO_H_ (high-density groups) (Table 1). Also, each 75th percentile in the distributions of the TIL numbers in ascending order were determined as cutoffs (75th percentile cutoffs) for dichotomisation of the variables, that is, 75th percentile or a percentile less than 75 were considered as low-density groups and a percentile greater than 75 as high-density groups for each marker. Each 75th percentile was 452, 191, and 92 per area of 0.44 mm^2^, for CD3^+^, CD8^+^, and CD45RO^+^ TILs, respectively. We also grouped cases using combined variables, that is, as high or low for CD3+CD8, CD3+CD45RO, CD8+CD45RO, and CD3+CD8+CD45RO (refer to Table 1 for a complete list). We then compared groups dichotomised about means and about 75th percentile cutoffs. Because comparisons of the high- and low-density groups of CD20^+^, TIL using various cutoffs failed to show any significant difference in terms of clinicopathologic characteristics or survival, no detailed data on CD20^+^ TIL is presented.

### Correlation between TIL density and clinicopathologic characteristics

In a total of 220 gastric cancer patients, median age was 57 years (range, 18–80 years), and 70.9%. were male. The clinical and pathological characteristics of the cases grouped by TIL density with mean value cutoffs are summarised in Table 2. TIL density showed no correlation with either tumour invasion depth or the presence of lymphatic invasion, but it was found to be significantly associated with the presence of regional lymph node metastasis; CD3_H_, CD8_H_, and CD45RO_H_ groups showed a lower frequency of lymph node metastasis than the corresponding low-density groups. Considering this observation, we assumed that TIL density would predict lymph node metastasis in gastric cancer.

### TIL as a predictor of regional lymph node metastasis

According to univariate logistic regression model, an advanced T stage, the presence of lymphatic invasion, and low densities of CD3^+^, CD8^+^, or CD45RO^+^ TILs were significantly associated with presence of regional lymph node metastasis regardless of the grouping method (mean value cutoff or 75th percentile cutoff). Low-density groups by combination of variables also showed significant relationships with the presence of lymph node metastasis. Multivariate logistic regression analysis, after adjusting for tumour invasion (T stage) and lymphatic invasion, demonstrated that densities of CD3^+^, CD8^+^, and CD45RO^+^ TILs were independent predictors of lymph node metastasis. As expected, combined analysis of variables showed similar results (Tables 3 and 4).

### TIL as a predictor of patient survival

**Figure 2:**
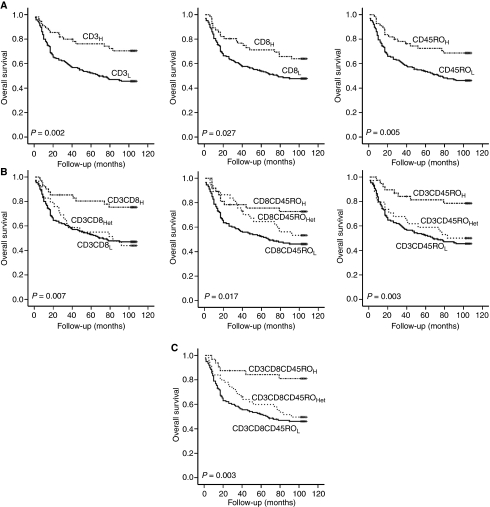
Kaplan–Meier survival plots for the 220 gastric cancer patients as a function of TIL density. (**A**) Significant differences in overall survival were found between the low- and high-density groups of CD3, CD8, and CD45RO, by the log-rank tests. (**B**, **C**) Survival differences were consistently found when analysing with diverse combined TIL variables (refer to Table 1).

Significant advantages for overall survival were found for the CD3_H_, CD8_H_, and CD45RO_H_ groups and for the high-density groups of combined TIL variables (refer to Table 1) according to 75th percentile cutoffs by Kaplan–Meier survival analysis (Figure 2). The analysis with variables according to mean value cutoffs showed similar results (data not shown). CD3_H_, CD8_H_, and CD45RO_H_ groups had mean survival times of 85.1±5.2, 81.0±5.3, and 83.1±5.3 months, respectively, as compared with only 63.0±3.5, 64.3±3.5, and 63.7±3.5 months in the corresponding low-density groups. Thus, the density of various TILs seems to be useful for defining a subgroup with unfavourable prognosis in gastric cancer.

Univariate Cox regression analysis for the prediction of overall survival also confirmed that the CD3_H_, CD8_H_, and CD45RO_H_ groups and the high-density groups of combined TIL variables had better survival than the corresponding low-density groups regardless of the grouping method (mean value cutoff or 75th percentile cutoff). In addition, densities of CD3^+^, CD8^+^ and CD45RO^+^ TILs and the combined TIL densities remained significant predictors of overall survival by multivariate Cox proportional hazard analysis, even after controlling for T stage, histologic classification (WHO), presence of lymph node metastasis, and presence of lymphatic invasion, which were all found to be of prognostic significance by univariate analysis (Tables 5 and 6).

### Two-fold cross-validation approach

For further validation of our results, we performed a two-fold cross-validation approach. In analyses using this approach, univariate and multivariate logistic regression analyses confirmed that densities of CD3^+^, CD8^+^, and CD45RO^+^ TILs were independent predictors of regional lymph node metastasis (Table 7). In univariate Cox regression analysis, CD3_H_, CD8_H_, and CD45RO_H_ groups were proved to have longer survival time than the corresponding low-density groups (Table 8). In multivariate Cox regression analysis, CD45RO^+^ TIL density remained a statistically significant predictor of overall survival, and CD8^+^ TIL density was associated with overall survival marginally, but CD3^+^ TlL density did not remain a significant predictor (Table 8).

## Discussion

**Figure 3:**
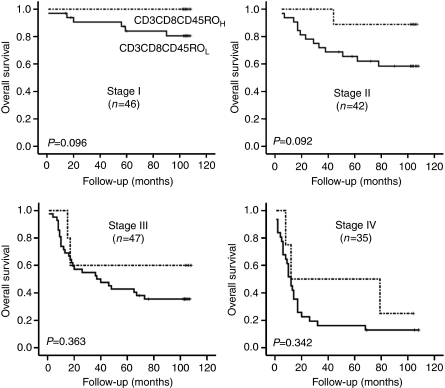
Kaplan–Meier survival analysis of TIL densities at different stages of the TNM classification (log-rank tests). High TIL density tended to be associated with longer overall survival within a given TNM stage. The results were from 75th percentile cutoffs.

The present study demonstrates that the type and density of TILs correlate with the clinical outcome after gastrectomy in gastric cancer. TIL was found to be an independent predictor of lymph node metastasis and an independent prognostic factor of patients' overall survival by multivariate analyses. In addition, we evaluated an effect of TIL density on survival at each stage of TNM classification. Overall survival of patients with a high TIL density tended to be longer than that of patients with a low TIL density within the same TNM stage, although differences were not statistically significant due to small numbers of cases enrolled in each stage (Figure 3). This finding suggests that the diversity of clinical outcomes shown by gastric cancer patients with the same TNM stage is due in part to differences in TIL density, which underlines the importance of TIL as a predictor of clinical outcome.

In this study, cases were classified into low or high TIL density groups using mean number or 75th percentile cutoffs, and the results were consistent regardless of the grouping method used. In addition, analyses using two-fold cross-validation approach strengthened our results, although it failed to show that CD3^+^ TIL density was an independent predictor of overall survival. Nevertheless, the counting method using image analysis software and the classification system used in this study would be difficult to use clinically. Thus, we suggest that a standard method be devised to measure TIL densities for clinical applications.

Recent studies on colorectal cancer have demonstrated that a high density of CD45RO^+^ TIL is correlated with the absence of lymphovascular invasion and with increased survival (Pages *et al*, 2005), and further that the densities of CD3^+^, CD8^+^, and CD45RO^+^ TILs are independent prognostic factors (Galon *et al*, 2006). In a study on ovarian cancer, it was found that the presence of intratumoral T cells was associated with improved clinical outcome (Zhang *et al*, 2003). Previous reports also show that hepatocellular and urothelial carcinoma patients with high numbers of CD8^+^ TIL within tumour tissues have better survival (Gao *et al*, 2007; Sharma *et al*, 2007). In terms of lymph node metastasis, Taylor *et al* (2007) reported that brisk TIL infiltration in tumour predicts sentinel lymph node metastasis in melanoma patients, and Piersma *et al* (2007) demonstrated that a high number of intraepithelial CD8^+^ TIL is associated with the absence of lymph node metastasis in uterine cervical cancer.

However, the prognostic role of tumour-infiltrating immune cells in patients of gastric cancer is largely unknown. Only a few reports have been issued on the association between tumour-infiltrating immune cells and the clinical outcome in gastric cancer; Ishigami *et al* (2000) reported that patients showing a high level of natural killer cell infiltration in tumour tissues have a better prognosis, and Maehara *et al* (1997) showed that a high density of dendritic cell infiltration is associated with the absence of lymph node metastasis. Ichihara *et al* (2003) reported that the population of regulatory T cells among the TILs of patients with advanced disease (*n*=8) is significantly higher than that among the TILs of patients with early disease (*n*=7). On the other hand, Fukuda *et al* (2002) found no significant difference in survival between patients with marked or slight TIL infiltration, which does not agree with our findings. However, they detected TILs by UCLH-1 immunostaining in 129 gastric cancer patients, classified cases into groups with marked or slight TIL infiltration, and did not determine TIL numbers. In this study of a large series of gastric cancers, we counted the number of tumour-infiltrating total T cells, cytotoxic T cells, memory T cells, and B cells using an image analyser, and for the first time demonstrated the prognostic importance of TIL in gastric cancer.

T-cell mediated adaptive immunity is considered to play a major role in anti-tumour immunity. In mouse models, it has been demonstrated that adaptive immunity prevents the development of tumours and inhibit tumour progression (Dunn *et al*, 2004). Consistent with these results, our findings show that high densities of immune cells related to adaptive immunity, that is, total T cells, cytotoxic T cells, and memory T cells, are associated with favourable survival, and indicate that adaptive immunity plays a role in the prevention of tumour progression. On the other hand, tumour-infiltrating B cells are found not to have impact on patient survival in this study. Previous reports have concluded that B lymphocytes and humoral immunity are associated with tumour progression (de Visser *et al*, 2006; Sabbatini and Odunsi, 2007), which is not contradicted by our findings.

According to our results, TIL density is correlated with the presence of lymph node metastasis but not with depth of tumour invasion. On the basis of this finding, we suspect that the prognostic role of TIL is mainly contributed to decreased metastatic potential. We suggest the following possible mechanisms how TIL act to reduce metastatic potential. First, clones with metastatic potential usually contain larger amounts of aberrantly expressed proteins, including proteins that contribute to metastasis, which may act as tumour-associated antigens. As a result, these clones are more likely to be destroyed by *in situ* immune reactions. Second, a high density of TIL means a healthy immune system, and therefore, immune reaction occurring in lymph node may also exert a proper function against tumour cells that have drained into lymph nodes in patients with high TIL densities. Third, tumour burden of metastatic foci in lymph node is less bulky than those of primary foci, and thus, metastatic foci are more likely to be susceptible to complete destruction by immune reaction.

In this study, we evaluated TIL distributions immunohistochemically, and although the majority of previous reports have also used this method, it has some limitations. First, the results obtained are dependent on the antibodies used. Second, various components of immune system such as interferon-*γ* (IFN-*γ*), IFN-*γ* receptor, transcription factor signal transducers and activators of transcription 1 (STAT1), perforin, and interleukins which can affect *in situ* immune reaction against tumour, and therefore, impact on patients' survival were not considered. Thus, additional studies are required to elucidate the expressional status of immune-related genes in tumour tissues.

Significant advances have been made in the field of cancer immunotherapy over the last decade, and many nonrandomised, phase II, immune-targeted trials have shown that immunotherapy can confer survival benefits, although these benefits were not confirmed in phase III trials (Rosenberg *et al*, 2004; Sabbatini and Odunsi, 2007). In gastric cancer, immunotherapy has not been shown to confer a survival benefit, although a clinical trial on postoperative adjuvant immunochemotherapy is currently in progress in Japan (Ueda *et al*, 2006). It is hoped that the observations made during this study will provide crucial information concerning the design of effective immunotherapies, and aid the identification of patients suitable for immunotherapy.

In conclusion, we elucidated the prognostic implications of the density of various types of TIL in gastric cancer. Moreover, our results suggest that adaptive immunity does act to prevent tumour progression. We believe that our findings can help predict clinical outcome and define patient subgroups with an unfavourable prognosis in gastric cancer. Furthermore, our observation will be useful for selecting patients that are more likely to benefit from the promising adjuvant immunotherapy.

## Figures and Tables

**Table 1:** Classification of the gastric cancers according to TIL density

**Variables**	**Mean value cutoff (cases, *N*)**	**75th percentile cutoff (cases, *N*)**
CD3_L_/CD3_H_	142/78	165/55
CD8_L_/CD8_H_	164/56	164/56
CD45RO_L_/CD45RO_H_	155/65	165/55
		
*Combination of variables*		
CD3_L_CD8 _L_/CD3CD8_Het_/CD3_H_CD8_H_	135/36/49	150/29/41
CD3_L_CD45RO_L_/CD3CD45RO_Het_/CD3_H_CD45RO_H_	131/35/54	148/34/38
CD8_L_CD45RO_L_/CD8CD45RO_Het_/CD8_H_CD45RO_H_	140/39/41	146/37/37
CD3_L_CD8_L_CD45RO_L_/ CD3CD8CD45RO_Het_/CD3_H_CD8_H_CD45RO_H_	125/55/40	138/50/32

H=high density group; Het=mixture of low and high density group for different markers; L=low density group.

**Table 2:** Correlation between TIL density and clinicopathologic characteristics in the 220 gastric cancers

	**CD3^+^ TIL**			**CD8^+^ TIL**			**CD45RO^+^ TIL**		
**Characteristics**	**Low**	**High**	*P*-value*	**Low**	**High**	*P*-value*	**Low**	**High**	*P*-value*
*Age (years)*			0.026			0.250			0.167
<66	108	69		129	48		121	56	
⩾66	34	9		35	8		34	9	
									
*Gender*			0.023			0.109			0.315
Male	108	48		121	35		113	43	
Female	34	30		43	21		42	22	
									
*WHO classification*			0.148			0.008			0.118
WD	9	3		11	1		9	3	
MD	50	23		58	15		53	20	
PD	58	44		65	37		65	37	
Mucinous	9	1		9	1		10	0	
SRC	16	7		21	2		18	5	
									
*Lymphatic invasion*			0.143			0.590			0.446
Absent	88	56		109	35		99	45	
Present	54	22		55	21		56	20	
									
*Tumour stage*			0.095			0.924			0.366
T1/2	98	62		119	41		110	50	
T3/4	44	16		45	15		45	15	
									
*LN metastasis*			<0.001			0.012			<0.001
Absent	24	31		34	21		28	27	
Present	118	47		130	35		127	38	
									
*TNM stage*			0.001			0.250			0.018
I	32	35		44	23		38	29	
II	32	21		41	12		38	15	
III	44	11		44	11		45	10	
IV	34	11		35	10		34	11	

LN=lymph node; MD=moderately differentiated tubular adenocarcinoma; Mucinous=mucinous adenocarcinoma; PD=poorly differentiated tubular adenocarcinoma; SRC=signet-ring cell carcinoma; WD=well differentiated tubular adenocarcinoma.

The results were according to the density groups of TIL with mean value cutoffs.

^*^χ^2^ test was used as a statistical method.

**Table 3:** Univariate and multivariate logistic regression models for predictors of regional lymph node metastasis (results from mean value cutoffs)

	**Univariate analysis**			**Multivariate analysis** a		
**Variables**	**OR**	**95% CI**	***P*-value**	**OR**	**95% CI**	***P*-value**
CD3^b^	0.308	0.164–0.580	<0.001	0.350	0.175–0.701	0.003
CD8^b^	0.436	0.225–0.843	0.014	0.325	0.150–0.707	0.005
CD45RO^b^	0.310	0.163–0.589	<0.001	0.302	0.147–0.622	0.001
CD3CD8^c^	0.307	0.148–0.640	0.002	0.277	0.122–0.632	0.002
CD3CD45RO^c^	0.234	0.116–0.473	<0.001	0.236	0.107–0.523	<0.001
CD8CD45RO^c^	0.292	0.136–0.625	0.002	0.260	0.111–0.608	0.002
CD3CD8CD45RO^d^	0.258	0.117–0.567	0.001	0.257	0.108–0.614	0.028

CI=confidence interval; OR=odds ratio.

The results were according to the density groups of TIL with mean value cutoffs.

Univariate and multivariate logistic regression models were used as statistical methods.

a Tumour invasion (T stage) and presence of lymphatic invasion were adopted as covariates in each multivariate analysis.

b high *vs* low.

c high/high *vs* low/low.

d high/high/high *vs* low/low/low.

**Table 4:** Univariate and multivariate logistic regression models for predictors of regional lymph node metastasis (results from 75th percentile cutoffs)

	**Univariate analysis**			**Multivariate analysis** a		
**Variables**	**OR**	**95% CI**	***P*-value**	**OR**	**95% CI**	***P*-value**
CD3^b^	0.375	0.194–0.726	0.004	0.425	0.204–0.885	0.022
CD8^b^	0.436	0.225–0.843	0.014	0.325	0.150–0.707	0.005
CD45RO^b^	0.420	0.217–0.815	0.010	0.402	0.190–0.850	0.017
CD3CD8^c^	0.338	0.161–0.710	0.004	0.318	0.138–0.734	0.007
CD3CD45RO^c^	0.321	0.149–0.689	0.004	0.337	0.143–0.793	0.013
CD8CD45RO^c^	0.400	0.180–0.888	0.024	0.354	0.146–0.860	0.022
CD3CD8CD45RO^d^	0.369	0.160–0.851	0.019	0.397	0.160–0.988	0.047

CI=confidence interval; OR=odds ratio.

The results were according to the density groups of TIL with 75th percentile cutoffs.

Univariate and multivariate logistic regression models were used as statistical methods.

a Tumour invasion (T stage) and presence of lymphatic invasion were adopted as covariates in each multivariate analysis.

b High *vs* low.

c High/high *vs* low/low.

d High/high/high *vs* low/low/low.

**Table 5:** Univariate and multivariate cox proportional hazards models for the predictors of overall survival (results from mean value cutoffs)

	**Univariate analysis**			**Multivariate analysis** a		
**Variables**	**HR**	**95% CI**	***P*-value**	**HR**	**95% CI**	***P*-value**
CD3^b^	0.627	0.410–0.627	0.031	0.813	0.523–1.264	0.358
CD8^b^	0.583	0.358–0.950	0.030	0.574	0.347–0.949	0.031
CD45RO^b^	0.530	0.331–0.850	0.008	0.612	0.377–0.995	0.048
CD3CD8^c^	0.522	0.303–0.900	0.019	0.572	0.325–1.010	0.054
CD3CD45RO^c^	0.441	0.252–0.772	0.004	0.565	0.317–1.009	0.054
CD8CD45RO^c^	0.416	0.221–0.785	0.007	0.436	0.227–0.838	0.013
CD3CD8CD45RO^d^	0.393	0.202–0.766	0.006	0.471	0.236–0.937	0.032

CI=confidence interval; HR=hazard ratio.

The results were according to the density groups of TIL with mean value cutoffs.

Univariate and multivariate Cox proportional hazards models were used as statistical methods.

a Tumour stage (T stage), histologic classification (WHO), presence of lymph node metastasis, and presence of lymphatic invasion which were proved to have a prognostic significance in univariate analysis were adopted as covariates in each multivariate analysis.

b High *vs* low.

c High/high *vs* low/low.

d High/high/high *vs* low/low/low.

**Table 6:** Univariate and multivariate cox proportional hazard models for the predictors of overall survival (results from 75th percentile cutoffs)

	**Univariate analysis**			**Multivariate analysis** a		
**Variables**	**HR**	**95% CI**	***P*-value**	**HR**	**95% CI**	***P*-value**
CD3^b^	0.446	0.261–0.760	0.003	0.549	0.317–0.951	0.032
CD8^b^	0.583	0.358–0.950	0.030	0.574	0.347–0.949	0.031
CD45RO^b^	0.483	0.287–0.813	0.006	0.507	0.298–0.862	0.012
CD3CD8^c^	0.373	0.193–0.721	0.003	0.403	0.205–0.795	0.009
CD3CD45RO^c^	0.305	0.147–0.632	0.001	0.337	0.161–0.708	0.004
CD8CD45RO^c^	0.418	0.216–0.809	0.010	0.413	0.211–0.812	0.010
CD3CD8CD45RO^d^	0.271	0.118–0.624	0.002	0.282	0.121–0.658	0.003

CI=confidence interval; HR=hazard ratio.

The results were according to the density groups of TIL with 75th percentile cutoffs.

Univariate and multivariate Cox proportional hazards models were used as statistical methods.

a Tumour stage (T stage), histologic classification (WHO), presence of lymph node metastasis, and presence of lymphatic invasion which were proved to have prognostic significance in univariate analysis were adopted as covariates in each multivariate analysis.

b High *vs* low.

c High/high *vs* low/low.

d High/high/high *vs* low/low/low.

**Table 7:** Univariate and multivariate logistic regression models for predictors of regional lymph node metastasis (results from two-fold cross-validation approach)

	**Univariate analysis**			**Multivariate analysis** a		
**Variables**	**OR**	**95% CI**	***P*-value**	**OR**	**95% CI**	***P*-value**
CD3^b^	0.42	0.211–0.818	0.011	0.45	0.213–0.935	0.033
CD8^b^	0.32	0.158–0.630	0.001	0.34	0.162–0.723	0.005
CD45RO^b^	0.29	0.153–0.554	<0.001	0.27	0.128–0.554	<0.001
CD3CD8^c^	0.28	0.124–0.614	0.001	0.31	0.128–0.729	0.003
CD3CD45RO^c^	0.26	0.123–0.527	<0.001	0.25	0.108–0.563	<0.001
CD8CD45RO^c^	0.21	0.097–0.459	0.001	0.20	0.085–0.479	0.001
CD3CD8CD45RO^d^	0.20	0.083–0.463	<0.001	0.20	0.077–0.518	<0.001

CI=confidence interval; OR=odds ratio.

Univariate and multivariate logistic regression models were used as statistical methods.

a Tumour invasion (T stage) and presence of lymphatic invasion were adopted as covariates in each multivariate analysis.

b high *vs* low.

c high/high *vs* low/low.

d high/high/high *vs* low/low/low.

**Table 8:** Univariate and multivariate cox proportional hazards models for the predictors of overall survival (results from two-fold cross-validation approach)

	**Univariate analysis**			**Multivariate analysis** a		
**Variables**	**HR**	**95% CI**	***P*-value**	**HR**	**95% CI**	***P*-value**
CD3^b^	0.57	0.343–0.935	0.026	0.67	0.404–1.123	0.130
CD8^b^	0.59	0.374–0.938	0.026	0.64	0.408–1.003	0.052
CD45RO^b^	0.30	0.151–0.594	0.001	0.42	0.209–0.843	0.015
CD3CD8^c^	0.49	0.288–0.837	0.009	0.56	0.324–0.964	0.037
CD3CD45RO^c^	0.34	0.162–0.691	0.003	0.44	0.211–0.923	0.030
CD8CD45RO^c^	0.28	0.134–0.592	0.001	0.36	0.170–0.768	0.008
CD3CD8CD45RO^d^	0.31	0.148–0.656	0.002	0.39	0.185–0.836	0.015

CI=confidence interval; HR=hazard ratio.

Univariate and multivariate Cox proportional hazard models were used as statistical methods.

a Tumour stage (T stage), histologic classification (WHO), presence of lymph node metastasis, and presence of lymphatic invasion which were proved to have prognostic significance in univariate analysis were adopted as covariates in each multivariate analysis.

b high *vs* low.

c high/high *vs* low/low.

d high/high/high *vs* low/low/low.
